# What can we learn from the effect of trihexyphenidyl on motor fluctuations during continuous levodopa-carbidopa intestinal gel infusion (LCIG)? – First documented case

**DOI:** 10.1016/j.prdoa.2020.100071

**Published:** 2020-08-26

**Authors:** Shin-ichiro Kubo, Ken Nakamura, Yoshimasa Tada, Nobuyuki Kashio, Shinya Yamashita

**Affiliations:** a Department of Neurology, Eisei Hospital, Tokyo, Japan; b Department of Gastroenterology, Eisei Hospital, Tokyo, Japan

**Keywords:** Motor fluctuations, LCIG, Afternoon off, Anticholinergics, Parkinson's disease

## Abstract

Motor fluctuations can be seen even during treatment with continuous levodopa-carbidopa intestinal gel infusion (LCIG). We report on a middle-aged man with advanced Parkinson's disease (PD) on LCIG in which motor fluctuations have been improved with an anticholinergic. To the best of our knowledge, there have been no previous LCIG cases reported with motor fluctuations responding to non-dopaminergic agent, which might reveal some clues to its pathophysiology. Long-term oral levodopa treatment is associated with development of potentially disabling motor complications including motor fluctuations and dyskinesias in the majority of patients with PD. It has been suggested that motor complications are related to the nonphysiological restoration of brain dopamine with intermittent administration of standard oral levodopa. LCIG significantly reduces “off” time and increases “on” time without dyskinesia in comparison to standard oral levodopa through consistent plasma concentration of levodopa to restore brain dopamine in a more physiological manner. However, it has been reported that PD patients on LCIG often worsen during the afternoon hours, even with stable plasma concentration of levodopa. This raises the possibility that additional factors to dopamine deficiency could play a role in occurrence of motor fluctuations. Here we offer a hypothesis that altered cholinergic signaling could also be involved in the pathophysiology of motor fluctuations, based on our clinical evidence that anticholinergic drug has eliminated motor fluctuations during LCIG in a patient with PD. Further studies for non-dopaminergic along with dopaminergic signaling may be needed to better understand the pathophysiological basis of motor complications in PD.

## Highlights

• Motor fluctuations can occur during the afternoon hours in Parkinson's patients even on LCIG.
• Anticholinergic agent could be an option for treating the motor fluctuations during LCIG.
• Altered cholinergic signaling might be involved in occurrence of motor fluctuations.

Levodopa remains the gold standard treatment for Parkinson's disease (PD). With long-term oral levodopa therapy, however, the majority of PD patients begin to experience potentially disabling motor complications, including motor fluctuations and dyskinesias. Growing evidence suggests that these are related to the nonphysiological restoration of brain dopamine with intermittent administration of standard oral levodopa [1]. It has been hypothesized that continuous delivery of levodopa could restore brain dopamine in a more physiological manner and prevent or reverse motor complications. This concept was supported by a double-blind trial demonstrating that continuous levodopa-carbidopa intestinal gel infusion (LCIG) is associated with significantly reduced “off” time and increased “on” time without dyskinesia in comparison to optimized standard oral levodopa [2]. In theory, LCIG generates sustained stimulation of striatal neurons through consistent plasma concentration of levodopa by providing a continuous infusion of levodopa and carbidopa by means of a portable pump and percutaneous endoscopic gastrojejunostomy tube [3]. Strangely, it has been reported that PD patients on LCIG are often troubled by motor fluctuations during the afternoon hours, even with stable plasma concentration of levodopa [4]. Here we offer a hypothesis that cholinergic signaling might also play a role in the pathophysiology of motor fluctuations, based on the authors' clinical experience as described below and review of the literature.

**Fig. 1:**
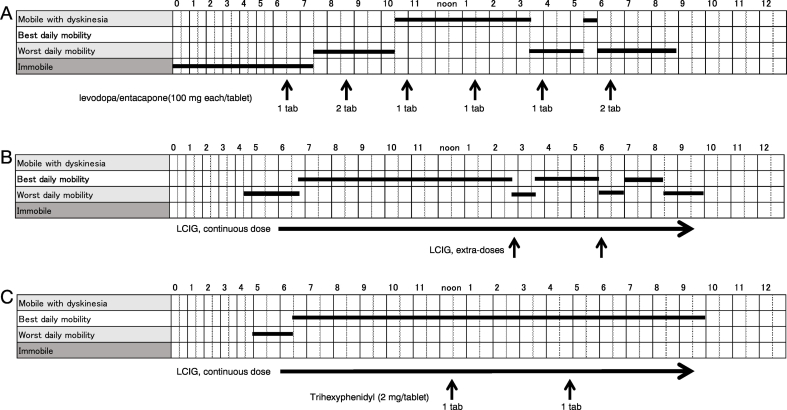
The clinical effect of levodopa-carbidopa intestinal gel (LCIG) treatment based on the patient's motor diaries. A: Before LCIG treatment, the patient fluctuates between poor “off” states and “on” states with dyskinesias on oral LD dosing every 2.5 h, 6 times a day (arrows). B: After commencement of LCIG treatment, the increased favorable “on” condition is the most important gain from LCIG treatment. Extra-doses of LCIG (arrows) are required for the afternoon “off”. C: Oral administration of trihexyphenidyl (arrows) prevents the occurrence of the afternoon “off”.

A 52-year-old man presented with gradual right-sided upper extremity rest tremor, bradykinesia and rigidity at the age of 43. He then visited the outpatient clinic of our hospital and was diagnosed as PD. He started on treatment with levodopa/benserazide (300 mg/day) and pramipexole (1.875 mg/day) which led to a dramatic improvement for four years. In the following years, due to progressive worsening of motor fluctuations, levodopa/benserazide was gradually increased up to 800 mg/day and associated treatment with entacapone (800 mg/day) was added. Pramipexole increased up to 3 mg/day was withdrawn because of impulse control disorder (gambling). At age 52, his motor condition was characterized by severe “off” period with related right upper and bilateral lower extremities rest tremor, sometimes alternated with peak-dose dyskinesias. His scores on UPDRS part III in the “on” and “off” states were 9 and 50, Hoehn and Yahr stages were II and IV, respectively. His cognitive screening was normal (MMSE = 30/30). Nine years after disease onset, his collective “off” time of 8 h, alternating with peak-dose dyskinesias (Fig. 1A), prompted his recruitment into the LCIG treatment. A significant reduction of the “off” periods was obtained through LCIG titration of the morning dose to 6 ml (120 mg of levodopa), of the continuous dose to 3.2 ml (64 mg) per hour per 16 h a day (total dose of 1024 mg) and of the extra-doses to 2.5 ml (50 mg) as needed. However, during the days following LCIG initiation, he experienced a decreasing response to medication consistently in the afternoon hours (Fig. 1B). Although the extra-doses alleviated the afternoon “off” symptoms comprising the severe rest tremor in right upper and bilateral lower extremities along with generalized bradykinesia (Fig. 1B), he continued to complain an unsatisfactory control of the motor fluctuations. Rotigotine (9 mg/day) was effective for management of nocturnal “off” during the period when the pump was turned off, but not for the afternoon “off”. As the afternoon “off” typically occurred at around 2–3 pm (Fig. 1B), based on the possibility that levodopa competes with dietary large neutral amino acids from lunch for intestinal absorption [5] and/or transport across the blood-brain barrier [6], we attempted to manage his afternoon “off” by a protein-redistribution diet [7], but it was unsuccessful. We then added trihexyphenidyl (2 mg, twice a day at noon and 5 pm) as an empirical therapeutic trial for reduction in parkinsonian tremor [8], exerting a preventative effect against not only the tremor but also the afternoon “off” per se (Fig. 1C).

## Beyond *wearing-off*: additional factors involved in the underlying mechanism of motor fluctuations

When standard oral levodopa therapy is initiated, the benefit from levodopa is usually sustained, with general improvement throughout the day and no dose-timing variations. However, long-term oral levodopa treatment is associated with the development of motor fluctuations. At first, fluctuations take the form of *wearing-off* (also known as end-of-dose deterioration), which is defined as a return of parkinsonian symptoms in less than 4 h after the last dose [9]. Gradually, the duration of benefit shortens further and the “off” state becomes more profound. In some patients these fluctuations become more abrupt in onset and random in timing; the condition is then the “on-off” effect and cannot be related to the timing of the levodopa intake. Occasionally, motor fluctuations take the form of *weak response at end of day* [9], which may coincide with the afternoon “off” in the present case. Clinicopharmacological studies have demonstrated different patterns of motor fluctuations with different underlying mechanisms, which include well-characterized peripheral factors related to levodopa pharmacokinetics, gastrointestinal transit, absorption, and transport as well as striatal pharmacodynamic changes that are less well understood [6]. The short half-life of levodopa in plasma of approximately 90 min is associated with peaks and valleys of plasma levels. Absorption of levodopa only begins in the duodenum and is thus dependent on gastric emptying, such that reduced gastric emptying after a meal or as part of the gastrointestinal symptoms of PD can translate into a delayed onset of drug effect. Intestinal absorption of levodopa occurs through active transport mediated by a specific transporter for large neutral amino acids, such that competition with neutral amino acids from the diet can reduce plasma levels and the clinical effect from a dose [5]. A similar competition can also occur at the blood–brain barrier [6]. Although the pathophysiology of the afternoon “off” during LCIG remains poorly understood [4], most of the peripheral factors are supposed to be resolved with LCIG on the basis of its mechanism of action. Altered threshold for levodopa responses during the day has been suggested as the possible mechanism of the afternoon “off”, consistent with the fact that patients on LCIG suffering from the afternoon “off”, including our case, usually require either extra doses or higher infusion rates during the latter part of the day [4]. The nature of the threshold also remains to be clarified, while the striatal pharmacodynamic changes could play a role [4,6]. Indeed, motor fluctuations are thought to be related to alterations in the striatal dopaminoceptive medium spiny GABAergic neurons and their synaptic connections with other striatal interneurons and cortical afferents that provide glutamatergic input [9]. Therefore, the preventative effect of trihexyphenidyl against the afternoon “off” in our case imply a potential role of the striatal cholinergic interneurons in the pathophysiology of motor fluctuations, given that trihexyphenidyl, an M1 muscarinic receptor antagonist, would exert its anti-parkinsonian effects by reducing the increased striatal cholinergic tone in PD [[10], [11], [12]]. Recent study using mouse model with parkinsonian-like motor deficits have also suggested that increased cholinergic transmission via M1 muscarinic receptors of the dorsal striatum plays a pivotal role in the occurrence of motor symptoms in PD [13].

It is important to acknowledge that the present report has a number of limitations that should be addressed. Although in our case the LCIG titration of the morning as well as continuous dose was determined just below the levels evoking dyskinesias, higher doses might have eliminated the afternoon “off”. Not only trihexyphenidyl but also the rest of known anti-PD drugs such as selegiline and amantadine could also be efficacious in the prevention of the afternoon “off” during LCIG. Further studies would be needed to establish a direct association between pharmacodynamic effect of anticholinergics and motor fluctuations.

In conclusion, these preliminary observations would serve the purpose for generating new hypothesis that altered cholinergic signaling in addition to dopamine deficiency also contributes to occurrence at least in part of motor fluctuations in PD, while there seems to be almost no place for anticholinergics, which have been used for the treatment of PD for a long period of time and currently known to associate with an increased risk of dementia, in the era of levodopa and device-aided therapies for PD [14,15].

## Ethical compliance statement

The authors confirm that the approval of an institutional review board was not required for this work. Written consent to publish all shown materials was obtained from the patient in Japanese.

## Funding sources

This report did not receive any specific grant from funding agencies in the public, commercial, or not-for-profit sectors.

## CRediT authorship contribution statement

SK: Study conception, study design, interpretation of the data, writing of the first draft, and review of the manuscript.

All other authors: Study conception, interpretation of the data, and review of the manuscript.

## Declaration of competing interest

**T**he authors declare that there are no conflict of interest relevant to this work. This report did not receive any specific grant from funding agencies in the public, commercial, or not-for-profit sectors.
